# C9orf72 Proteins Regulate Autophagy and Undergo Autophagosomal or Proteasomal Degradation in a Cell Type-Dependent Manner

**DOI:** 10.3390/cells8101233

**Published:** 2019-10-10

**Authors:** Stina Leskelä, Nadine Huber, Hannah Rostalski, Teemu Natunen, Anne M. Remes, Mari Takalo, Mikko Hiltunen, Annakaisa Haapasalo

**Affiliations:** 1 A. I. Virtanen Institute for Molecular Sciences, University of Eastern Finland, Neulaniementie 2, 70211 Kuopio, Finland; stina.leskela@uef.fi (S.L.); nadine.huber@uef.fi (N.H.); hannah.rostalski@uef.fi (H.R.); 2 Institute of Biomedicine, Yliopistonranta 1E, University of Eastern Finland, 70211 Kuopio, Finland; teemu.natunen@uef.fi (T.N.); mari.takalo@uef.fi (M.T.); mikko.hiltunen@uef.fi (M.H.); 3 Unit of Clinical Neuroscience, Neurology, University of Oulu, P.O. Box 8000, University of Oulu, 90014 Oulu, Finland; anne.remes@oulu.fi; 4 MRC Oulu, Oulu University Hospital, P.O. Box 8000, University of Oulu, 90014 Oulu, Finland

**Keywords:** amyotrophic lateral sclerosis, autophagy, *C9orf72*, frontotemporal dementia, proteasomal degradation, ubiquitin-proteasome system

## Abstract

Dysfunctional autophagy or ubiquitin-proteasome system (UPS) are suggested to underlie abnormal protein aggregation in neurodegenerative diseases. Frontotemporal dementia (FTD) and amyotrophic lateral sclerosis (ALS)-associated *C9orf72* is implicated in autophagy, but whether it activates or inhibits autophagy is partially controversial. Here, we utilized knockdown or overexpression of *C9orf72* in mouse N2a neuroblastoma cells or cultured neurons to elucidate the potential role of C9orf72 proteins in autophagy and UPS. Induction of autophagy in *C9orf72* knockdown N2a cells led to decreased LC3BI to LC3BII conversion, p62 degradation, and formation of LC3-containing autophagosomes, suggesting compromised autophagy. Proteasomal activity was slightly decreased. No changes in autophagy nor proteasomal activity in C9orf72-overexpressing N2a cells were observed. However, in these cells, autophagy induction by serum starvation or rapamycin led to significantly decreased C9orf72 levels. The decreased levels of C9orf72 in serum-starved N2a cells were restored by the proteasomal inhibitor lactacystin, but not by the autophagy inhibitor bafilomycin A1 (BafA1) treatment. These data suggest that C9orf72 undergoes proteasomal degradation in N2a cells during autophagy. Lactacystin significantly elevated C9orf72 levels in N2a cells and neurons, further suggesting UPS-mediated regulation. In rapamycin and BafA1-treated neurons, C9orf72 levels were significantly increased. Altogether, these findings corroborate the previously suggested regulatory role for C9orf72 in autophagy and suggest cell type-dependent regulation of C9orf72 levels via UPS and/or autophagy.

## 1. Introduction

Frontotemporal dementia (FTD), the most common clinical phenotype of frontotemporal lobar degeneration (FTLD), is the third most common cause of early onset dementia in individuals under 65 years of age [1]. FTLD is clinically, neuropathologically, and genetically a heterogeneous group of neurodegenerative syndromes, and interestingly, it shares a partially overlapping genetic and molecular pathological background with amyotrophic lateral sclerosis (ALS) [2,3]. The most common genetic cause underlying both FTD and ALS is the GGGGCC-hexanucleotide repeat expansion (HRE) in the *C9orf72* gene [4,5,6]. The pathological mechanisms of the HRE underlying neurodegeneration are controversial, but are suggested to involve haploinsufficiency, leading to a decreased expression of the normal *C9orf72* gene products (loss-of-function), as well as formation and accumulation of toxic RNA foci and dipeptide repeat (DPR) proteins that are directly generated from the expanded repeat (gain-of-toxic-function) [7,8,9,10]. Even though there is substantial evidence indicating that the main pathological mechanisms underlying HRE-associated FTD and ALS are related to gain-of-toxic-function, haploinsufficiency has also been suggested to contribute to the disease pathogenesis. Thus, neurodegeneration in HRE-linked FTD and ALS could involve co-operation between gain-of-toxic-function and loss-of-function mechanisms [11]. The normal physiological functions of the C9orf72 proteins, which may be influenced by the haploinsufficiency, are not yet well known. The *C9orf72* gene produces three different transcript variants, which in humans are translated to two different protein isoforms, the long isoform A (~50 kDa) and the short isoform B (~25 kDa) [5]. Isoform B has been recently implicated in nucleo-cytoplasmic transport [12], while the isoform A contains a differentially expressed in normal and neoplastic cells (DENN) domain and thus is suggested to act as a guanosine exchange factor (GEF) for Rab-GTPases [13,14]. Accumulating experimental evidence indicates that the C9orf72 isoform A interacts with, and possibly activates, multiple different Rab-GTPases, such as Rab1, Rab3, Rab5, Rab7, Rab8, Rab10, Rab11, Rab13, Rab15, Rab29, and Rab39 [10,15,16,17,18,19], although the interaction seems to depend on the cell type as the expression of Rab-GTPases might display tissue specificity [20]. Thus, by regulating GDP/GTP exchange and subsequent activation of Rab-GTPases, the C9orf72 isoform A is suggested to regulate vesicular trafficking in the endosomal-lysosomal and autophagosomal-lysosomal pathways [13,15,21].

Autophagy and the ubiquitin-proteasome system (UPS) are essential pathways controlling proteostasis in cells, especially during stress conditions, such as those prevailing in diseased brain. These pathways are in charge of degrading unfolded, misfolded, or aggregated proteins. Neurons, as non-dividing cells with long axons and dendrites, are especially vulnerable to alterations in proteostasis [22]. In fact, defects in autophagy and UPS-mediated protein degradation pathways are suggested to contribute to the pathogenesis of many neurodegenerative diseases, including FTD and ALS [23].

In macroautophagy, hereafter referred to as autophagy, proteins are guided to degradation through autophagy receptor proteins, such as sequestosome 1 (p62/SQSTM1, hereafter p62). Autophagy can be induced by multiple environmental stimuli, such as nutrient deprivation, which initiates autophagic processes in order to provide a supply of metabolites for vital cellular functions, or by accumulation of misfolded or aggregated proteins [24,25]. Proteins are selected for degradation by ubiquitination and conjugated to an adaptor molecule, such as p62, which targets them to the double-membrane phagophore by binding to a membrane-bound receptor protein (e.g., LC3BII) on its inner surface. The expanding ends of the phagophore membrane ultimately fuse to create the autophagosome, which in the later phases of autophagy fuses with a lysosome to initiate degradation of its contents [26]. In the UPS, the proteins are guided to degradation also by ubiquitination to their lysine residues. The poly-ubiquitinated proteins are then targeted to the proteasome, where they are degraded to smaller peptides and amino acids, which can be further re-used in protein synthesis [23]. The UPS and autophagy-mediated protein degradation pathways are not mutually exclusive, but rather are interlinked and co-operate to maintain proteostasis in cells [23,27].

In *C9orf72* HRE-associated FTD or ALS, the DPR proteins have been reported to hinder or block protein degradation through the UPS, emphasizing that the *C9orf72* HRE causes toxicity by gain-of-toxic-function mechanisms [28,29]. On the other hand, *C9orf72* loss-of-function due to haploinsufficiency has been suggested to lead to reduced autophagic degradation and subsequent accumulation of the DPR proteins, implicating that concomitant loss of *C9orf72* function further exacerbates the effects of the gain-of-toxic-function mechanisms [30]. However, the potential role of the loss-of-function mechanisms in contributing to the toxicity of the *C9orf72* HRE is still controversial. Recent studies have suggested that the C9orf72 protein isoform A regulates autophagy, but the reports have been partially controversial on whether reduced levels of *C9orf72* lead to impaired or enhanced autophagy and at which steps on the autophagic pathway these effects take place [10,15,16,17,18,19,31,32,33]. In the present study, we have examined the role of C9orf72 in autophagy and UPS-mediated protein degradation pathways in neuronal cells and primary neurons. In addition, we have elucidated the yet-unknown regulation of the C9orf72 protein itself through autophagy and UPS-mediated protein degradation.

## 2. Materials and Methods

### 2.1. shRNA and cDNA Constructs

To knockdown endogenous *C9orf72*, we used SMARTvector Lentiviral Mouse 3110043O21Rik shRNA containing mEF1a promoter and TurboRFP tag (Dharmacon, Lafayette, CO, USA, target sequence ATTATGTTAAGATCGCCCT, targets all transcripts). SMARTvector Non-targeting Control 1 with mEF1a promoter and TurboRFP (Dharmacon) was used as a control. To overexpress the C9orf72 protein isoform A, a cDNA construct encoding human C9orf72 isoform A containing a C-terminal green fluorescent protein (GFP) tag (C9-A-GFP) was purchased from Origene (Rockville, MD, USA) and used as previously [34]. Empty plasmid (pcDNA3.1, Thermo Scientific, Carlsbad, CA, USA) was used as a control in the cDNA transfections.

### 2.2. Secondary Cell Culture, Transfection, and Treatments

N2a mouse neuroblastoma cells were cultured in Dulbecco’s modified Eagle’s medium (DMEM, Lonza, Walkersville, MD, USA) supplemented with 10% fetal bovine serum (FBS, Gibco, Paisley, UK), 2 mM L-glutamine, 100 U/mL penicillin, and 100 µg/mL streptomycin (Lonza, Walkersville, MD, USA) at +37 °C and 5% CO_2_. A total of four µg of the plasmid DNA and 10 μL Lipofectamine 2000 reagent (Thermo Scientific, Carlsbad, CA, USA) were used per transfection of 400,000 cells according to the manufacturer’s instructions (scaled down proportionally for smaller cell amounts) to knockdown *C9orf72* or to overexpress the C9orf72 protein isoform A. Fresh media were added 6 h post transfection when overexpressing the C9orf72 protein isoform A or after 24 h when using the shRNA to knockdown *C9orf72*. Cells were collected 48 (overexpression) or 72 h (knockdown) post transfection for analyses. 

To induce autophagy, serum starvation (ST) by culturing the cells in a medium without added FBS or treatment with 200 nM of rapamycin (Sigma, St. Louis, MO, USA) was used overnight. To assess autophagic flux, serum-starved cells were co-treated with 300 nM bafilomycin A1 (BafA1, Sigma, St. Louis, MO, USA) for the last 6 h to block the late phase of autophagy. In rapamycin-treated cells, the cells were co-treated with 200 nM rapamycin and 50 nM BafA1 overnight. To block protein degradation through the UPS, 10 µM lactacystin (Enzo Life Sciences, Farmingdale, NY, USA) was added overnight [15]. Dimethyl sulfoxide (DMSO, Hybri-Max, Sigma, St. Louis, MO, USA) was used as a vehicle control.

### 2.3. Immunofluorescence Studies

In immunofluorescence experiments, glass coverslips were coated with 1:100 Matrigel GFR Basement Membrane Matrix (Corning, Bedford, MA, USA) for 1 h at room temperature (RT). N2a cells (ATCC) were simultaneously reverse co-transfected with either control or *C9orf72* shRNA (3/4 of the total plasmid amount) and GFP-tagged LC3 construct (1/4 of the total plasmid amount). The GFP-LC3 plasmid was a kind gift from Prof. Kai Kaarniranta (UEF). Fresh media were changed 24 h after transfection and the cells were treated as described above. At 72 h post-transfection, the cells were fixed in 4% paraformaldehyde (PFA, Thermo Scientific, Rockford, IL, USA) for 10 min at RT and mounted with Vectashield Vibrance antifade mounting medium containing 4′,6-diamidino-2-phenylindole (DAPI, Vector Laboratories, Burlingame, CA, USA) to counterstain the nuclei. Images were taken with a LSM700 (ZEISS) confocal microscope and analyzed with ImageJ (Fiji, NIH). Quantification of the LC3-positive puncta was performed by first selecting shRNA-transfected cells (turboRFP-positive) for the analysis. After subtracting the background, the image was filtered by using the blur option, and correct threshold settings were chosen to ensure that the background signal was not detected as puncta but also that the real signal from the puncta was not lost. A particle analysis tool was used to quantify the number of puncta in each cell.

### 2.4. Primary Mouse Cortical Cell Culture, Virus Vector Transduction and Treatments

Primary mouse cortical cell cultures were established as previously [35]. Briefly, cortical cells were harvested from embryonic day 18 mouse embryos. A single-cell suspension was prepared from the dissected cortices by trypsin digestion and trituration. The cells were plated (400,000/well) on poly-d-lysine (PDL, Sigma, St. Louis, MO, USA)-coated 24-well plates in serum-free Neurobasal medium (Gibco, Grand Island, NY, USA) supplemented with 1 × B27 (Gibco, Grand Island, NY, USA), 2 mM l-glutamine, 100 U/mL penicillin, and 100 μg/mL streptomycin. Half of the medium was changed every fourth day to feed the neurons. The cultures contain 90–95% of cortical neurons and 5–10% of astrocytes and other cells [36]. The neurons were matured for 11 days before the C9-A-GFP lentivirus-mediated transduction using 26 multiplicity of infection (MOI). The medium containing the viral particles was removed 24 h after transduction. The cells were cultured for 72 h after transduction before sample collection.

To induce autophagy in the neuronal cultures, overnight treatment with 200 nM of rapamycin was used. To block the late phase of autophagy, BafA1 was used at a 50 nM concentration simultaneously with autophagy induction [37] to measure the autophagic flux or separately for 6 h at 300 nM concentration to assess the basal autophagy. To block protein degradation through the UPS, treatment with 10 µM lactacystin was used overnight. DMSO was used as a vehicle control.

### 2.5. Protein Extraction from Cells and Western Blotting

Proteins were extracted by scraping into T-PER, tissue protein extraction buffer, (Thermo Scientific, Rockford, IL, USA) supplemented with 1:100 protease and phosphatase inhibitors (Thermo Scientific, Rockford, IL, USA). The T-PER-soluble protein fractions were cleared by centrifugation of the cell lysates for 10 min at 10,000× *g* at +4 °C. The resulting pellet was resuspended to 1% sodium dodecyl sulfate (SDS, Sigma, St. Louis, MO, USA) extraction buffer (10 mM Tris-HCl (Sigma, St. Louis, MO, USA), 2 mM EDTA, (Sigma, St. Louis, MO, USA)) to collect the T-PER-insoluble protein fraction. Protein concentrations were measured using the Pierce BCA Protein Assay Kit (Thermo Scientific, Rockford, IL, USA), and 15–50 μg of proteins were separated on sodium dodecyl sulfate–polyacrylamide gel electrophoresis (SDS-PAGE) gels (NuPAGE Novex 4–12% Bis-Tris midi, Thermo Scientific, Carlsbad, CA, USA) for 1 h 45 min at 100 V. The proteins were transferred onto polyvinylidene fluoride (PVDF) membrane (Trans-Blot^®^ Turbo™ Midi PVDF, Bio-Rad, Hercules, CA, USA) using Trans-Blot^®^ Turbo™ Transfer System (Bio-Rad, 25 V, 30 min). After the transfer, unspecific binding sites on the membranes were blocked with 5% non-fat dry milk or bovine serum albumin (BSA, Sigma, St. Louis, MO, USA) in 1 x Tris-buffered saline with 0.1% Tween 20 (Sigma, St. Louis, MO, USA) (TBST) for 1 h at RT. The protein bands were detected by incubating the membrane with protein-specific primary antibodies overnight at +4 °C and appropriate horse radish peroxidase-conjugated secondary (ECL™, anti-mouse IgG or anti-rabbit IgG, GE Healthcare, Buckinghamshire, UK, anti-mouse IgM, Thermo Scientific, Rockford, IL, USA) antibodies for 1 h at RT. The proteins were detected using enhanced chemiluminescence (ECL) detection reagents (Amersham Biosciences, GE Healthcare, Buckinghamshire, UK) and ChemiDoc™ XRS+ System (Bio-Rad). The intensities of the detected protein bands were quantified with Image Lab™ software (5.2.1, Bio-Rad) and normalized to those of glyceraldehyde-3-phosphate dehydrogenase (GAPDH) or β-actin (loading controls). The membrane was stripped with a stripping buffer (Thermo Scientific, Rockford, IL, USA) for 10 min at RT, after which it was washed in 1 x TBST and reprobed with other antibodies. The following primary antibodies were used: anti-C9orf72 (1:500; Proteintech, Rosemont, IL, USA, 22637-1-AP); anti-SQSTM1/p62 (1:1000; Cell Signaling Technology (CST, Danvers, MA, USA 5114)); anti-LC3B (1:3000; Abcam, Cambridge, UK, ab51520); anti-poly-ubiquitinated proteins (FK1, 1:1000, Enzo Life Sciences, Farmingdale, NY, USA, BML-PW8805-0500); anti-LAMP2A (1:1000, Abcam, Cambridge, UK, ab18528); anti-beta-actin (1:1000, Abcam, Cambridge, UK, ab8226), and anti-GAPDH (1:5000, Abcam, Cambridge, UK, ab8245). The data are shown as % of the protein level compared to that in the control plasmid or control shRNA-transfected cells, or vehicle-treated cells (set to 100%).

### 2.6. Proteasomal Activity Measurement

Proteasomal chymotrypsin-like activity was measured with a UBPBio kit (Aurora, CO, USA, J4110) or Abcam proteasomal activity kit (Cambridge, UK, ab107921) according to the kit instructions. Briefly, proteasomes from N2a cells transfected with control or *C9orf72* shRNA or with pcDNA or C9-A-GFP constructs were extracted using a 0.5% NP-40 (Sigma, St. Louis, MO, USA) lysis buffer (prepared in distilled water) and centrifugation at 16,000× *g* for 20 min at +4 °C. The protein concentrations were measured using a Pierce BCA Protein Assay Kit. Ten µl of each proteasome lysate was incubated with the proteasomal substrate Succ-LLVY-AMC without or with the proteasomal inhibitor MG-132 (negative control) in two separate wells. The resulting fluorescence, indicating proteasomal activity, was measured at excitation/emission wavelength of 360 nm/460 nm, respectively, with an Infinite M200 (Tecan) plate reader. The specific activity of only the proteasomes, excluding the activity of other proteases present in the sample, was acquired by subtracting the fluorescence value of the corresponding MG-132-treated sample from the total fluorescence value in the sample without MG-132 treatment. The values were further normalized to the protein concentration of each sample and shown as % of control shRNA or pcDNA-transfected samples (set to 100%).

### 2.7. Statistical Analyses

The data are shown as mean % ± standard error of mean (SEM) of control plasmid-, control shRNA-transfected or vehicle-treated cells as indicated. Statistical analyses were performed using GraphPad Prism5 (5.03). One-way analysis of variance (ANOVA) followed by Newman‒Keuls post-hoc test or Mann-Whitney U test were used to test the statistical significance between sample groups. The level of statistical significance was defined as *p* ≤ 0.05.

## 3. Results

Previous studies have suggested that the C9orf72 isoform A is a regulator of autophagy. However, whether knockdown of *C9orf72* leads to enhanced or decreased autophagy is currently unclear and may depend on the cell type or on how the autophagy is modulated [10,15,16,17,18,19,31,32]. Furthermore, it is not completely clear if the site of action of the C9orf72 isoform A is in the early or later phases or both in the autophagic pathway [16,32,33]. Whether the C9orf72 proteins regulate the function of the UPS and whether they themselves are regulated by autophagy or UPS have not been studied before. Here, we have examined the effects of the knockdown or overexpression of C9orf72 protein isoform A on autophagy and UPS in N2a mouse neuroblastoma cells and mouse primary cortical neurons and investigated the regulation of C9orf72 protein isoform A itself via these protein degradation pathways.

### 3.1. C9orf72 Knockdown Leads to Decreased Autophagy Induction in N2a Cells

**Figure 1:**
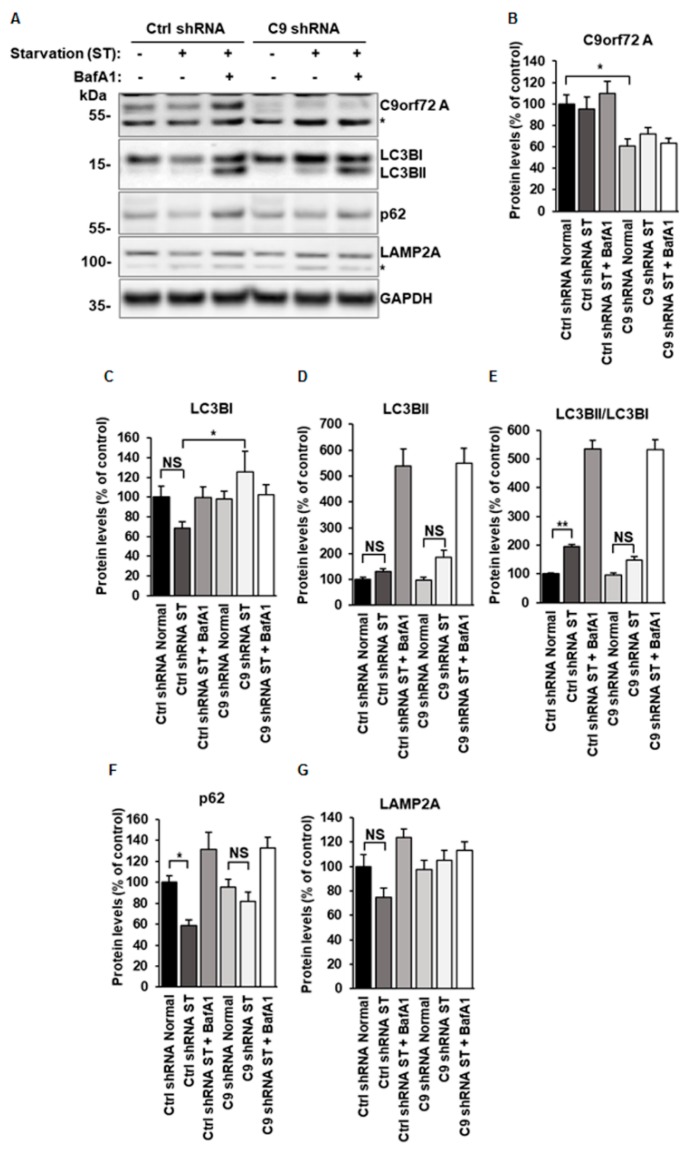
Knockdown of C9orf72 in N2a cells leads to decreased LC3BI to LC3BII conversion and p62 degradation during serum starvation-induced autophagy. (**A**) A representative Western blot of the total protein lysates of N2a cells transfected with control (Ctrl) or C9orf72 (C9) shRNA showing the levels of the endogenous C9orf72 isoform A, LC3BI, LC3BII, p62, LAMP2A, and GAPDH (loading control) under normal growth conditions, after serum starvation (ST) or ST and bafilomycin A1 (BafA1) treatment. Overnight ST leads to autophagy induction as indicated by the conversion of LC3BI to LC3BII and the decrease in p62 levels in control shRNA-transfected cells but not in C9orf72 shRNA-transfected cells. The levels of p62 are restored and LC3BII robustly accumulates upon 300 nM BafA1 treatment for 6 h. Lysosomal LAMP2A levels did not show statistically significant alterations. Molecular weight markers are indicated on the left as kDa. An unspecific band, detected by the C9orf72 antibody and not affected by the shRNA, is indicated by an asterisk (*). Quantification of (**B**) the endogenous levels of C9orf72 isoform A, indicating an approximately 40% decrease in C9orf72 shRNA-transfected cells as compared to cells transfected with control shRNA, (**C**) LC3BI levels, (**D**) LC3BII levels, (**E**) ratio of LC3BII/LC3BI, (**F**) p62 levels, and (**G**) LAMP2A (unspecific band is indicated by *) levels. The protein levels were normalized to GAPDH levels in each sample. Data are shown as mean % ± SEM of the protein levels compared to those in the control cells. *n* = 9 from three independent experiments, except in p62 quantification, Ctrl shRNA Normal, *n* = 8 and LAMP2A quantifications, Ctrl shRNA ST *n* = 8; one-way ANOVA, Newman‒Keuls, * *p* ≤ 0.05, ** *p* ≤ 0.01.

During autophagy induction, phosphatidylethanolamine (PE) is conjugated with cytosolic LC3BI to form a membrane-bound lipidated LC3BII. Increased levels of LC3BII can therefore be used as a marker of autophagy induction and the number of autophagosomes present in the cells. Moreover, decreased levels of p62, a known autophagy receptor and substrate, reflect enhanced autophagic degradation [25,38]. Comparison of these factors can be used to help to distinguish between increased or decreased autophagy induction or autophagosomal clearance [25,38]. In N2a cells, 72-h shRNA treatment led to approximately a 40% decrease in the C9orf72 protein isoform A levels (Figure 1A,B). Endogenous expression of the isoform B was not detectable in the N2a cells. Autophagy was induced by overnight serum starvation and BafA1 was used to block the fusion of autophagosomes with lysosomes and thus the later phases of the autophagosomal degradation pathway [25,38]. In the control shRNA-transfected N2a cells, the LC3BII/LC3BI ratio significantly increased due to the conversion of LC3BI to LC3BII upon serum starvation (Figure 1C–E). Interestingly, in *C9orf72* knockdown cells, the levels of LC3BI were significantly higher than those in the control shRNA-transfected cells upon autophagy induction (Figure 1C). In addition, the ratio of LC3BII/LC3BI did not significantly increase in *C9orf72* knockdown cells similarly to control shRNA-transfected cells upon autophagy induction (Figure 1E). BafA1 treatment led to a similar strong accumulation of especially LC3BII and therefore increased LC3BII/LC3BI ratio in all the cells (Figure 1C–E).

The p62 levels significantly decreased upon induction of autophagy in the control shRNA-transfected cells, as expected. In the *C9orf72* knockdown cells, however, the decrease in p62 levels was not evident upon serum starvation-induced autophagy, and the levels remained similar to the cells cultured in the normal medium. p62 levels similarly accumulated after the BafA1 treatment in control and *C9orf72* shRNA-transfected cells (Figure 1F). To determine whether knockdown of *C9orf72* has an effect on the lysosomes, the immunoblot was probed with an antibody against LAMP2A, a lysosomal protein. There were no significant changes in LAMP2A levels in *C9orf72* shRNA-transfected cells as compared to the control shRNA-transfected cells or between the different treatments (Figure 1A,G).

**Figure 2:**
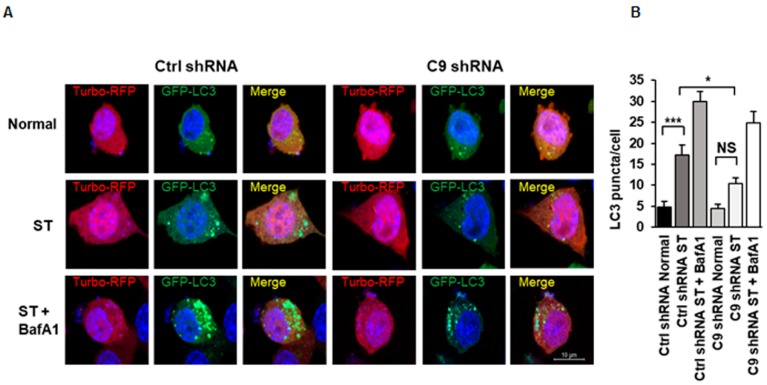
Knockdown of C9orf72 in N2a cells leads to decreased autophagosome formation during serum starvation-induced autophagy. (**A**) Representative fluorescent microscope images of the control or C9orf72 shRNA and GFP-LC3 plasmid co-transfected cells in normal growth medium, upon ST-induced autophagy or combined treatment with ST and BafA1. Scale bar: 10 µm. (**B**) Quantification of the LC3 puncta with ImageJ software from two independent experiments. Four coverslips were analyzed/each treatment group. Number of the analyzed cells in total: Ctrl shRNA Normal 21, C9 shRNA Normal 14, Ctrl shRNA ST 22, C9 shRNA ST 28, Ctrl shRNA ST + BafA1 48, C9 shRNA ST + BafA1 22. Data are shown as mean ± SEM, one-way ANOVA, Newman‒Keuls, * *p* ≤ 0.05, *** *p* ≤ 0.001.

Immunofluorescent studies (Figure 2A) revealed significantly fewer GFP-LC3 puncta after serum starvation-induced autophagy in the *C9orf72* knockdown cells compared to the control shRNA-transfected cells (Figure 2B), indicating decreased autophagosome formation. BafA1 treatment resulted in increased number of the LC3 puncta in both control and *C9orf72* shRNA-transfected cells. These findings, in agreement with previous reports, support the idea that knockdown of *C9orf72* leads to a defective autophagy induction [16,17,18,31].

### 3.2. Overexpression of C9orf72 Isoform A Does Not Affect Autophagy, But Its Levels are Decreased after Induction of Autophagy in N2a Cells

**Figure 3:**
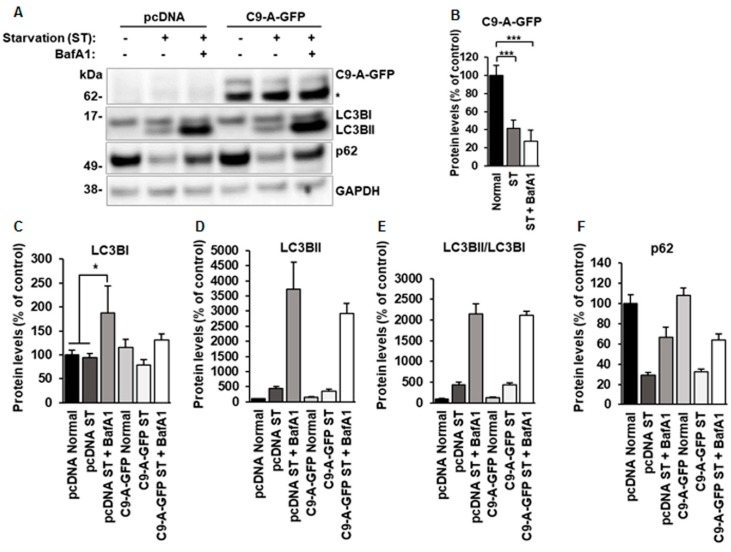
N2a cells overexpressing the C9orf72 isoform A do not show alterations in autophagy but display decreased C9orf72 isoform A levels after serum starvation. (**A**) A representative Western blot of the total protein lysates of N2a cells transfected with the control pcDNA or C9orf72 isoform A-GFP (C9-A-GFP) plasmids showing the levels of C9-A-GFP, LC3BI, LC3BII, p62, and GAPDH (loading control) under normal growth conditions, after serum starvation (ST) or ST and BafA1 treatment. ST overnight leads to similarly increased LC3BII and decreased p62 levels in both the control and the C9-A-GFP overexpressing cells. The levels of p62 and LC3BII accumulate in a similar manner after 300 nM BafA1 treatment for 6 h in the pcDNA and C9-A-GFP-transfected cells. C9-A-GFP levels are decreased after ST treatment and are not restored after BafA1 treatment. Molecular weight markers are indicated on the left as kDa. An unspecific band detected by the C9orf72 antibody is indicated by an asterisk (*). Quantification of (**B**) C9-A-GFP, (**C**) LC3BI, (**D**) LC3BII, (**E**) LC3BII/LC3BI ratio, and (**F**) p62 levels. Data are shown as mean % ± SEM of the protein levels compared to those in the control cells from three independent experiments. *n* = 8, except C9-A-GFP ST + BafA1, *n* = 5. One-way ANOVA, Newman‒Keuls, * *p* ≤ 0.05, *** *p* ≤ 0.001.

Next, we assessed whether overexpression of the C9orf72 protein isoform A affects autophagy in N2a cells. As the commercially available antibodies typically produce a high degree of unspecific binding, we transfected N2a cells with a cDNA construct encoding C9-A-GFP, to allow a reliable examination of the levels and effects of the C9orf72 isoform A, or an empty vector (pcDNA). The cells were serum-starved overnight and treated with BafA1 for 6 h before sample collection as before. No significant differences in the levels of LC3BI, LCBII, ratio of LC3BII/LC3BI, or the levels of p62 were detected in N2a cells overexpressing the C9orf72 isoform A (Figure 3A,C,F) as compared to control cells, suggesting that overexpression of C9orf72 protein isoform A does not influence autophagy. Moreover, in co-immunoprecipitation experiments, we did not detect interaction of the C9orf72 protein isoform A with LC3B or p62 under normal conditions or upon autophagy induction by serum starvation (data not shown).

**Figure 4:**
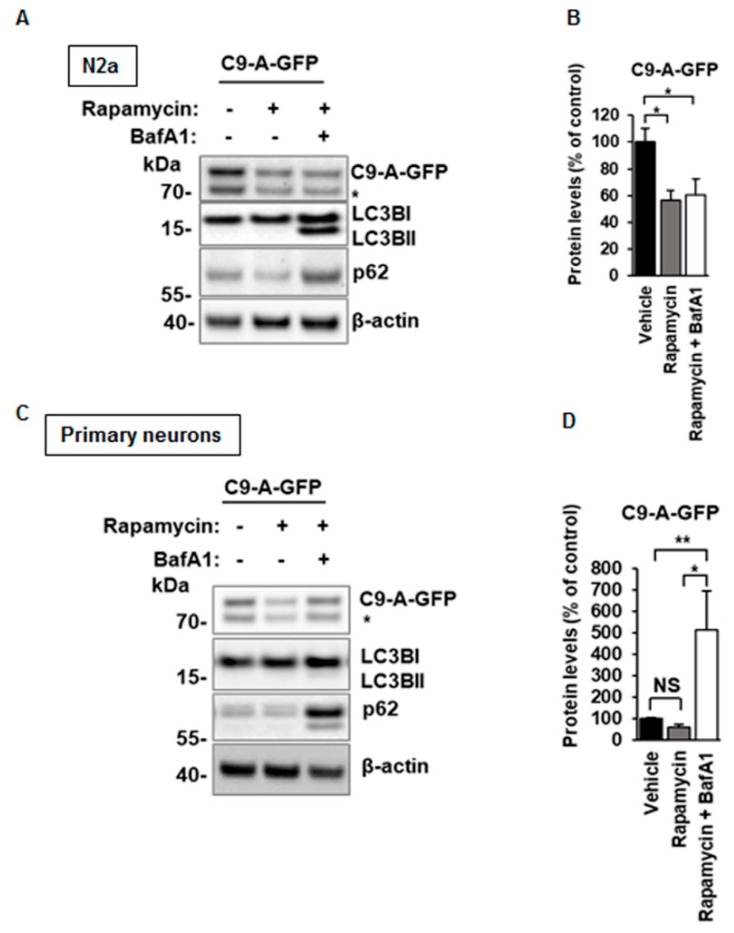
Induction of autophagy by rapamycin leads to decreased C9orf72 protein isoform A levels, which are not restored in N2a cells but show a significant increase in mouse cortical neurons upon BafA1 treatment. (**A**) A representative Western blot of the total protein lysates extracted from transfected N2a cells showing the levels of C9-A-GFP, LC3BI, LC3BII, p62, and β-actin (loading control). Treatment with 200 nM rapamycin ± 50 nM Bafilomycin A1 (BafA1) overnight leads to decreased levels of C9-A-GFP and the levels are not restored after BafA1 treatment. Molecular weight markers are indicated on the left as kDa. An unspecific band, detected by the C9orf72 antibody, is indicated by an asterisk (*). (**B**) Quantification of the C9-A-GFP protein levels in N2a cells normalized to those of β-actin. Data are shown as mean % ± SEM of the protein levels compared to those in the vehicle-treated cells from two independent experiments. *n* = 6, except C9-A-GFP rapamycin, *n* = 5, one-way ANOVA, Newman‒Keuls, * *p* ≤ 0.05. (**C**) A representative Western blot of total protein lysates extracted from lentivirus-transduced primary cortical neurons showing the levels of the C9-A-GFP, LC3BI, LC3BII, p62, and β-actin (loading control). Molecular weight markers are indicated on the left as kDa. An unspecific band detected by the C9orf72 antibody is indicated by an asterisk (*). (**D**) Quantifications of C9-A-GFP protein levels normalized to those of β-actin. Data are shown as mean % ± SEM of the protein levels compared to those in the vehicle treated cells from three independent experiments. *n* = 11, one-way ANOVA, Newman‒Keuls, * *p* ≤ 0.05, ** *p* ≤ 0.01.

Interestingly, a statistically significant decrease in the levels of the C9orf72 protein isoform A (Figure 3A,B) was detected after serum starvation-induced autophagy in N2a cells, suggesting that it might undergo autophagy-mediated degradation. However, when we further tested this hypothesis by blocking the autophagosomal degradation by BafA1, we did not detect increased C9orf72 protein isoform A levels (Figure 3A,B) in N2a cells in a similar manner to those of p62, a known autophagy substrate (Figure 3F). The C9orf72 isoform A levels were also significantly decreased when autophagy was induced by treatment of the N2a cells with rapamycin, which specifically targets the mammalian target of rapamycin complex 1 (mTORC1) (Figure 4A,B). Again, the levels of the C9orf72 isoform A were not restored after BafA1 treatment in rapamycin-treated cells, as would be expected if the proteins were regulated through autophagic degradation (Figure 4A,B). Together, the results from these experiments suggest that the decreased C9orf72 protein isoform A levels upon autophagy induction are not caused by their autophagy-mediated degradation in N2a cells.

In contrast to the N2a cells, however, a statistically significant increase in the levels of C9orf72 isoform A after rapamycin and BafA1 treatment in primary neurons was detected (Figure 4C,D), suggesting that in these cells, the C9orf72 protein isoform A may undergo autophagy-mediated degradation upon rapamycin-induced autophagy. These results imply differential regulation of the C9orf72 protein isoform A levels in different cell types upon modulation of autophagy.

### 3.3. C9orf72 Protein Isoform A Levels Are Increased after Proteasomal Inhibition in N2a Cells and Primary Neurons

**Figure 5:**
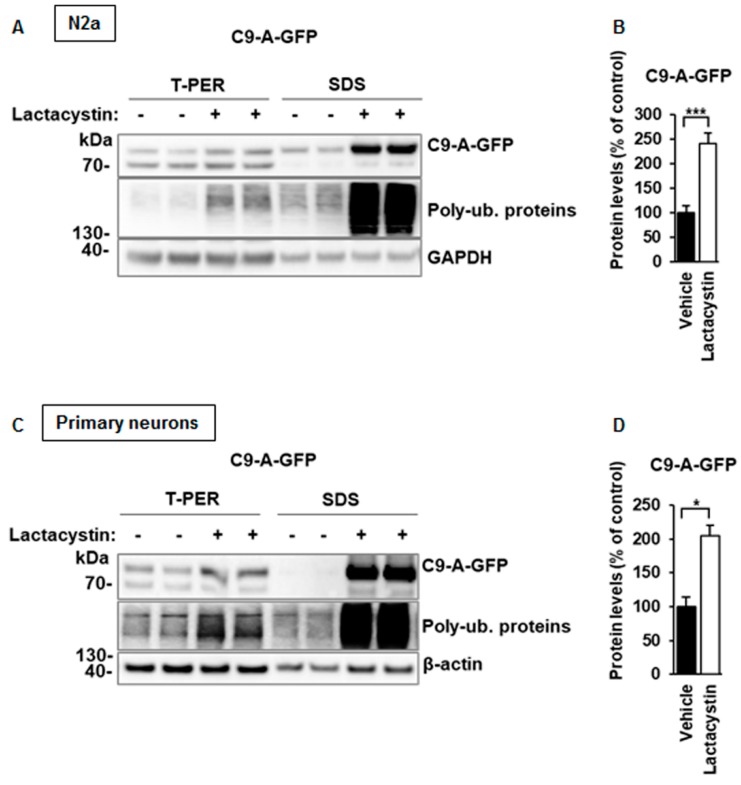
C9orf72 protein isoform A levels are elevated after proteasomal inhibition in N2a cells and mouse primary cortical neurons. (**A**) A representative Western blot of the total protein lysates extracted in T-PER and SDS extraction buffers from transfected N2a cells or (**C**) transduced mouse primary cortical neurons showing the levels of the C9-A-GFP, poly-ubiquitinated (Poly-Ub.) proteins, GAPDH or β-actin (loading control). Treatment with 10 µM lactacystin overnight leads to significantly increased levels of C9-A-GFP proteins and accumulation of poly-ubiquitinated proteins, especially to the SDS-soluble fraction in both N2a cells and cortical neurons. Molecular weight markers are indicated on the left as kDa. An unspecific band detected by the C9orf72 antibody is indicated by an asterisk (*). (**B**,**D**) Quantification of C9-A-GFP protein levels in the T-PER fraction of N2a cells or primary mouse cortical neurons normalized to those of GAPDH or β-actin. Data are shown as mean % ± SEM of the protein levels compared to those in the vehicle-treated cells from two independent experiments. *n* = 8 for all other samples, except n = 6 for C9-A-GFP-transduced neurons treated with vehicle, Mann‒Whitney U test, * *p* ≤ 0.05, *** *p* ≤ 0.001.

As we observed that the C9orf72 isoform A protein levels were decreased during autophagy, but not restored after blockage of autophagosomal degradation by BafA1 in N2a cells, we asked whether they could be regulated through the UPS. To this end, N2a cells transfected with the C9-A-GFP were treated overnight with the proteasomal inhibitor lactacystin. A significant increase in C9orf72 isoform A levels was observed (Figure 5A,B). In addition, C9orf72 protein isoform A accumulated in the SDS-soluble fraction, containing large amounts of accumulating poly-ubiquitinated proteins, upon lactacystin treatment (Figure 5A). Isoform A strongly accumulated in a similar manner in both T-PER- and SDS-soluble fractions and also in cultured mouse primary cortical neurons (Figure 5C,D). Taken together, these results suggest that C9orf72 proteins are regulated by the UPS in both N2a cells and mouse primary cortical neurons.

### 3.4. Decreased C9orf72 Protein Isoform A Levels in Serum-Starved N2a Cells Are Restored by Proteasomal Inhibition

**Figure 6:**
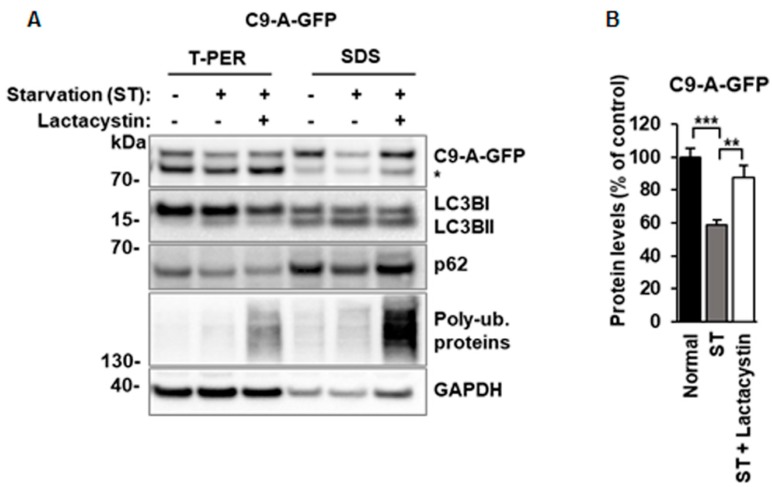
Decreased C9orf72 protein isoform A levels upon serum starvation-induced autophagy are restored by lactacystin treatment in N2a cells. (**A**) A representative Western blot of the total protein lysates extracted in T-PER and SDS extraction buffers from transfected N2a cells showing the levels of C9-A-GFP, LC3BI, LC3BII, p62, poly-ubiquitinated proteins (poly-Ub.), and GAPDH (loading control). Overnight serum starvation (ST) leads to decreased C9-A-GFP protein levels that are restored upon 10 µM lactacystin treatment. The levels of p62 also slightly increase, but LC3BI or II levels do not show major changes after lactacystin treatment in serum-starved cells. Molecular weight markers are indicated on the left as kDa. An unspecific band detected by the C9orf72 antibody is indicated by an asterisk (*). (**B**) Quantification of the C9-A-GFP protein levels normalized to those of GAPDH from two independent experiments. Data are shown as mean % ± SEM of the protein levels compared to those in the vehicle treated cells. *n* = 6 for all samples, one-way ANOVA, Newman‒Keuls, ** *p* ≤ 0.01, *** *p* ≤ 0.001.

We next tested the hypothesis whether the decrease in the C9orf72 protein isoform A levels during serum starvation-induced autophagy in N2a cells could be explained by its targeting to proteasomal degradation. Induction of autophagy by serum starvation in N2a cells again led to significantly decreased C9orf72 isoform A protein levels (Figure 6A,B). When the proteasomal degradation was blocked in these serum-starved cells using lactacystin, the C9orf72 protein isoform A levels were restored back to the basal levels (Figure 6A,B), suggesting that the decrease in the C9orf72 protein isoform A levels during autophagy is due to their proteasomal degradation in N2a cells.

### 3.5. Proteasomal Activity Is Slightly Decreased in N2a Cells Upon C9orf72 Knockdown but Not Changed After C9orf72 Protein Isoform A Overexpression

**Figure 7:**
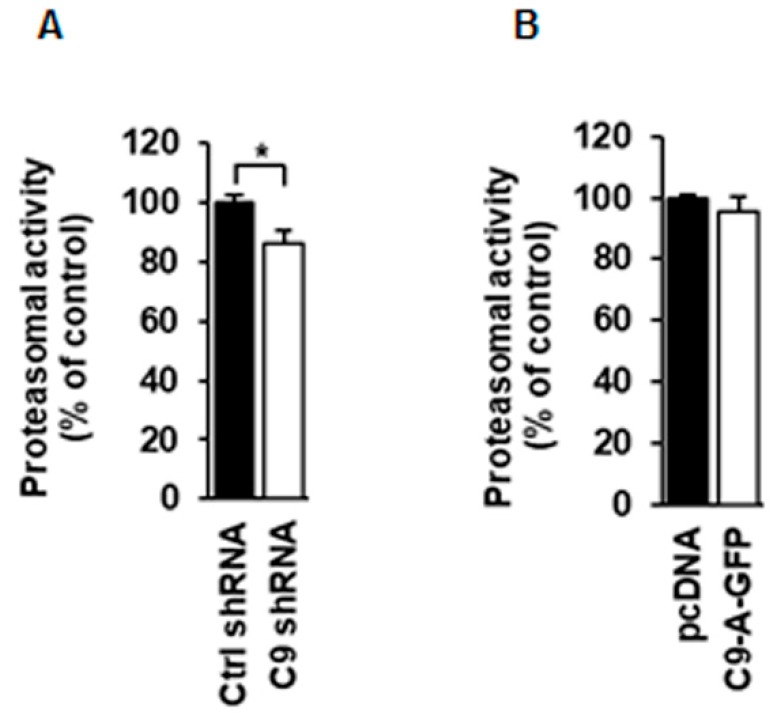
C9orf72 knockdown mildly decreases whereas overexpression of the C9orf72 isoform A does not affect the proteasomal activity in N2a cells. Quantification of proteasomal activity (**A**) in cells transfected with the control (Ctrl) or the C9orf72 (C9) shRNA. Data are shown as mean % ± SEM of the activity in control shRNA samples. n = 6 from two independent experiments, Mann‒Whitney U test, * *p* ≤ 0.05. (**B**) in cells overexpressing the C9-A-GFP. Data are shown as mean % ± SEM of the activity in pcDNA samples. *n* = 6 from two independent experiments, one-way ANOVA, Newman‒Keuls, not significant.

Previous studies by others have shown that C9orf72 interacts with ubiquilin 1 and 2 proteins, which are linked to protein degradation through the UPS [15]. Therefore, we next assessed the effects of the C9orf72 protein isoform A on proteasomal function. Here, we used both shRNA-mediated knockdown of endogenous *C9orf72* or overexpression of the C9orf72 protein isoform A in N2a cells and measured the proteasomal activity with the help of the fluorescent proteasomal substrate Succ-LLVY-AMC. Each extracted proteasome lysate from the transfected cells was incubated with the proteasomal substrate without or with the proteasomal inhibitor MG-132 (negative control) in two separate wells per each experiment. The specific activity of only the proteasomes, excluding the activity of other proteases present in the sample, was acquired by subtracting the fluorescence value of the corresponding MG-132-treated lysate from the total fluorescence value in the lysate without the MG-132 treatment. Proteasomal activity was slightly, but statistically significantly, decreased in *C9orf72* knockdown cells, whereas there was no significant difference in the proteasomal activity in cells overexpressing the C9orf72 isoform A compared to control cells (Figure 7A,B). As overexpression of C9orf72 isoform A did not have an effect on the proteasomal activity and the *C9orf72* knockdown effect was modest, these data imply that C9orf72 may not have a major regulatory role in UPS function, at least in N2a cells. 

### 3.6. C9orf72 Protein Isoform A Levels Are Unchanged After BafA1 Treatment in N2a Cells, but Decreased in Mouse Primary Cortical Neurons

**Figure 8:**
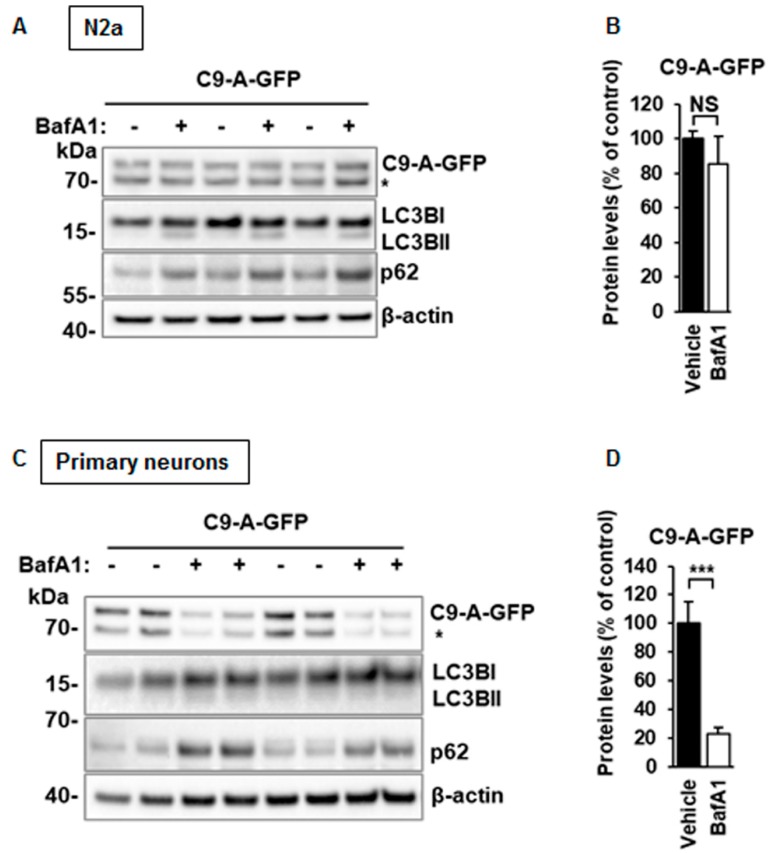
Blockade of the basal autophagy with BafA1 does not significantly change the C9orf72 protein isoform A levels in N2a cells but leads to its decreased levels in mouse primary cortical neurons. (**A**) A representative Western blot of the total protein lysates from transfected N2a cells showing the levels of C9-A-GFP, LC3BI, LC3BII, p62, and β-actin (loading control). Treatment with 300 nM Bafilomycin A1 (BafA1) for 6 h does not alter C9-A-GFP protein levels but increases p62 levels, as expected. Molecular weight markers are indicated on the left as kDa. An unspecific band detected by the C9orf72 antibody is indicated by an asterisk (*). (**B**) Quantification of the C9-A-GFP protein levels normalized to those of β-actin. Data are shown as mean % ± SEM of the protein levels compared to those in the vehicle treated cells from three independent experiments. *n* = 9, except in vehicle *n* = 8, Mann‒Whitney U test, not significant. (**C**) A representative Western blot of the total protein lysates from lentivirus-transduced mouse primary cortical neurons showing the levels of C9-A-GFP, LC3BI, LC3BII, p62, and β-actin (loading control). Treatment with 300 nM BafA1 for 6 h leads to significantly decreased levels of C9-A-GFP proteins, while p62 levels are increased, as expected. Molecular weight markers are indicated on the left as kDa. An unspecific band detected by the C9orf72 antibody is indicated by an asterisk (*). (**D**) Quantification of the C9-A-GFP protein levels normalized to those of β-actin. Data are shown as mean % ± SEM of the protein levels compared to those in the vehicle treated cells from two independent experiments. *n* = 8, except C9-A-GFP BafA1 *n* = 7, Mann‒Whitney U test, *** *p* ≤ 0.001.

Finally, we investigated whether the levels of the C9orf72 isoform A are regulated by autophagy in basal conditions without the induction of autophagy in N2a cells or primary cortical neurons. N2a cells overexpressing the C9orf72 isoform A were treated with BafA1 for 6 h. While the BafA1 treatment expectedly resulted in the accumulation of p62 and LC3BII, no significant changes were observed in the levels of C9orf72 protein isoform A in these conditions in N2a cells (Figure 8A,B). In contrast, in the mouse primary cortical neurons, the BafA1 treatment led to a significant decrease in the C9orf72 isoform A levels, while the p62 levels increased in a similar manner to those in N2a cells (Figure 8C,D). In neurons, BafA1 treatment did not majorly affect LC3BII levels. These data suggest that there might be cell type-specific differences in the basal autophagy and the regulation of C9orf72 levels.

## 4. Discussion

In the present study, we have examined the role of the C9orf72 protein isoform A in the autophagy and UPS-mediated protein degradation pathways and how it itself is regulated via these pathways in neuronal cells. The C9orf72 proteins mainly reside in the cytosol and they may also be secreted [15]. Additionally, the C9orf72 isoform A has been shown to localize in the lysosomes [10,15,31,33,39]. Previous studies have indicated that the C9orf72 isoform A forms a protein complex with Smith‒Magenis syndrome chromosome region candidate 8 (SMCR8) and WD repeat-containing protein 41 (WDR41) to regulate autophagy induction [16,18,31,33]. SMCR8, but not WDR41, protein levels are co-regulated with the levels of C9orf72 in cells [32] and a near-stoichiometric presence of these proteins has been suggested [18]. Moreover, the C9orf72 proteins have been suggested to interact with many proteins of the autophagy initiation complex, such as Unc-51 like autophagy activating kinase (ULK1), autophagy-related protein 13 (ATG13), and RB1-inducible coiled-coil protein 1 (RB1CC1/FIP200) [40], further suggesting a regulatory role in autophagy. The C9orf72 proteins have also been shown to interact with proteins like ubiquilin 1 and 2, which are linked to UPS functions [15].

The data on whether modulation of the C9orf72 levels leads to enhanced or impaired autophagy has been controversial and appear to depend on the model system used and how the autophagy is modulated. In some studies, reduction of the C9orf72 protein levels was shown to lead to impaired basal autophagy, whereas in other studies autophagy defects are detected only after induction of autophagy with for example torin 1 or rapamycin. Moreover, the phase of the autophagic pathway at which the C9orf72 proteins display their effects appears to vary in different studies [15,16,17,18,31,40].

Webster et al. [17] have reported increased autophagic activity in non-neuronal HeLa and HEK293 cells after C9orf72 overexpression. We did not detect alterations in autophagy upon overexpression of the C9orf72 isoform A in neuronal N2a cells. In contrast, *C9orf72* knockdown in N2a cells led to impaired autophagy, which is in accordance with several previous studies [16,17,18,31]. The C9orf72 isoform A has been suggested to represent the predominantly expressed isoform in mouse central nervous system (CNS) [10]. In line with this, we found that N2a cells do not express detectable levels of the isoform B. Therefore, we hypothesize that reduced levels of the C9orf72 isoform A underlie the observed defect in autophagy. The impairment in autophagy induction was suggested by the findings that knockdown of *C9orf72* led to impaired LC3BI to LC3BII conversion and degradation of p62, markers that can be used to assess autophagy initiation, in serum-starved N2a cells [38]. In addition, significantly fewer GFP-LC3-containing autophagosomes were detected in *C9orf72* knockdown N2a cells with serum starvation-induced autophagy when compared to control cells. Our data showed that the effect of the *C9orf72* knockdown on LC3BII/LC3BI ratio, as detected by Western blotting, was milder than that on the number of GFP-LC3-positive autophagosomal vesicles observed by immunofluorescence microscopy. It is possible that these differences might reflect a possibly deleterious effect of *C9orf72* knockdown on the trafficking of the autophagosomal vesicles, while the effect on autophagosome biogenesis might be more modest. Alternatively, these differences could be due to differential analysis of a total cell population in the Western blots vs. analysis of individual transfected cells in the immunofluorescence microscopy.

Furthermore, we did not observe any changes in lysosomal marker protein LAMP2A levels, implying that *C9orf72* knockdown may not majorly influence the late phases in the autophagosomal-lysosomal pathway in N2a cells. On the other hand, the finding that LAMP2A protein levels remained unchanged does not rule out possible morphological changes in the lysosomes or changes in the lysosome number in cells, which could be revealed by immunofluorescence studies. For example, Shi et al. [41] found no changes in LAMP2A levels in the lysosomal membrane fraction from *C9orf72* repeat expansion-carrying FTD patient-derived induced motor neurons, but immunofluorescence and electron microscopy studies revealed fewer lysosomes, which contained a higher concentration of LAMP2A proteins on their membrane surface. Altogether, these results corroborate the recent reports showing similarly impaired autophagy induction upon *C9orf72* deficiency in other cell types. Furthermore, our findings together with the previous studies support the idea that the haploinsufficiency, taking place in the FTD and ALS patients carrying the *C9orf72* HRE and resulting in the decreased C9orf72 levels, may contribute to defective autophagy and accumulation of, e.g., aggregated TDP-43 and DPR proteins, which represent the typical pathological hallmark changes in the CNS of the *C9orf72* HRE carriers. Our data further suggest that the C9orf72 proteins mainly regulate protein degradation via the autophagosomal pathway and do not play a major role in controlling the UPS in neuronal cells, as overexpression of the C9orf72 protein isoform A did not alter and the *C9orf72* knockdown only mildly decreased proteasomal activity in N2a cells.

An interesting novel finding was that in N2a cells, serum starvation- or rapamycin-induced autophagy led to significantly decreased levels of the C9orf72 isoform A. These levels were not restored after blocking the autophagosomal degradation by BafA1 treatment similarly to those of p62, a known autophagy substrate. Furthermore, blocking the basal autophagic degradation using BafA1 did not significantly change C9orf72 protein levels in N2a cells, while p62 levels increased as expected. Together, these data suggest that the C9orf72 proteins do not undergo autophagy-mediated degradation in N2a cells. The C9orf72 protein levels were previously shown to increase after BafA1 and the proteasomal inhibitor MG-132 treatment in HEK293 cells, implying that in these non-neuronal cells, C9orf72 levels could be regulated through both autophagy and UPS [32]. We also observed that blocking the proteasomal protein degradation using lactacystin resulted in the increased levels of the C9orf72 protein isoform A in N2a cells and primary cortical neurons. Moreover, the decreased C9orf72 protein isoform A levels upon serum starvation-induced autophagy in N2a cells were restored by treatment with lactacystin, suggesting that the C9orf72 protein isoform A could be guided to UPS-mediated degradation during autophagy. Given the suggested role of C9orf72 isoform A in promoting autophagy induction, these results may suggest a feedback loop between autophagy and the UPS to regulate the C9orf72 isoform A levels during autophagy and to maintain the balance in cellular proteostasis.

In mouse cortical neurons, in contrast to N2a cells, co-treatment with rapamycin and BafA1 led to significantly elevated levels of the C9orf72 isoform A. Moreover, while BafA1 treatment alone (without autophagy induction) did not influence the C9orf72 isoform A levels in N2a cells, it significantly decreased the C9orf72 isoform A levels in primary cortical neurons. The reason for this observation during basal autophagy in neurons currently remains unclear but may support the notion that there is a crosstalk between the autophagic and UPS-mediated degradation pathways and that upon blockade of autophagy, C9orf72 proteins may alternatively be targeted to proteasomal degradation. Collectively, our results suggest that the C9orf72 isoform A levels may be differentially regulated via UPS and/or autophagy depending on the cell type during basal conditions and upon different autophagic stimuli.

Taken together, the present data confirm the previously suggested role for the C9orf72 protein isoform A in the regulation of autophagy and underscore the key role for C9orf72 in controlling autophagy induction in neuronal cells. Moreover, to our knowledge, we show for the first time that the C9orf72 protein isoform A itself can undergo regulation through both autophagy and proteasomal degradation pathways and suggest that this regulation may differ depending on the cell type and prevailing conditions. However, these findings warrant further confirmation in other models, such as cells with stable *C9orf72* knockdown or expression.

## Abbreviations

ALS	Amyotrophic lateral sclerosis
BafA1	Bafilomycin A1
CNS	Central nervous system
C9FTD/ALS	Concomitant FTD/ALS caused by *C9orf72* hexanucleotide repeat expansion
*C9orf*72	Chromosome 9 open reading frame 72 gene
C9orf72	C9orf72 protein coded by *C9orf72*
DENN	Differentially expressed in normal and neoplastic cells
DPR	Dipeptide repeat
FTD	Frontotemporal dementia
FTLD	Frontotemporal lobar degeneration
GEF	Guanosine exchange factor
HRE	Hexanucleotide repeat expansion
LC3B	Microtubule-associated proteins 1A/1B light chain 3B
mTOR	Mammalian target of rapamycin
p62/SQSTMI	Sequestosome 1
PE	Phosphatidylethanolamine
SMCR8	Smith-Magenis syndrome chromosome region candidate 8
TDP-43	TAR DNA-binding protein 43
UPS	Ubiquitin-proteasome system
WDR41	WD repeat-containing protein 41
